# Multilocus sequence typing: genetic diversity in *Trypanosoma cruzi* I (TcI) isolates from Brazilian didelphids

**DOI:** 10.1186/s13071-018-2696-9

**Published:** 2018-02-22

**Authors:** Fabiola Roman, Alena M. Iñiguez, Matthew Yeo, Ana M. Jansen

**Affiliations:** 1 Laboratório de Biologia de Tripanosomatídeos, Instituto Oswaldo Cruz, Fundacao Oswaldo Cruz, Rio de Janeiro, RJ Brazil; 2 Department of Infectious and Tropical Diseases, London School of Hygiene and Tropical Medicine, London, UK

**Keywords:** *Trypanosoma cruzi*, Chagas disease, Multilocus sequence typing, MLST, Recombination, *T. cruzi* I, Genetic diversity

## Abstract

**Background:**

*Trypanosoma cruzi* is a protozoan parasite characterized by extensive genetic heterogeneity. There are currently six recognised, genetically distinct, monophyletic clades designated discrete typing units (DTUs). TcI has the broadest geographical range and most genetic diversity evidenced by a wide range of genetic markers applied to isolates spanning a vast geographical range across Latin America. However, little is known of the diversity of TcI that exists within sylvatic mammals across the geographical expanse of Brazil.

**Results:**

Twenty-nine sylvatic TcI isolates spanning multiple ecologically diverse biomes across Brazil were analyzed by the application of multilocus sequence typing (MLST) using four nuclear housekeeping genes. Results revealed extensive genetic diversity and also incongruence among individual gene trees. There was no association of intralineage genotype with geography or with any particular biome, with the exception of isolates from Caatinga that formed a single cluster. However, haplotypic analyses of *METIII* and *LYT1* constitutive markers provided evidence of recombination events in two isolates derived from *Didelphis marsupialis* and *D. albiventris*, respectively. For diversity studies all possible combinations of markers were assessed with the objective of selecting the combination of gene targets that are most resolutive using the minimum number of genes. A panel of just three gene fragments (*DHFR-TS*, *LYT1* and *METIII*) discriminated 26 out of 35 genotypes.

**Conclusions:**

These findings showed geographical association of genotypes clustering in Caatinga but more characteristically TcI genotypes widely distributed without specific association to geographical areas or biomes. Importantly, we detected the signature of recombination events at the nuclear level evidenced by haplotypic analysis and incongruence.

**Electronic supplementary material:**

The online version of this article (10.1186/s13071-018-2696-9) contains supplementary material, which is available to authorized users.

## Background

The protozoan parasite *Trypanosoma cruzi*, the causative agent of Chagas disease, is a vector-borne zoonosis transmitted by hematophagous triatomine bugs (Hemiptera: Reduviidae: Triatominae). They are maintained in the sylvatic environment by a wide range of mammalian hosts species and endemic from southern USA to southern Argentina [1, 2]. The primary route of infection in humans is contact with infected triatomine bug faeces that are deposited on the skin during the blood meal. Infection occurs when parasites enter mammal hosts through skin lesions, the insect bite wound or directly through the mucosa. Domiciliated infestation of triatomine bugs has not been reported from the Amazon basin but enzootic transmission from non-domiciliated adult triatomines is known to occur [3]. Moreover sporadic human infection by *T. cruzi* is re-emerging as a food-borne disease in areas that were not previously endemic for Chagas disease. In the Amazon, oral infection is associated primarily with the ingestion of infected açaí juice and bacaba juice [4–6]. Also, in Venezuela, an outbreak of over 100 cases of acute Chagas disease was caused by the ingestion of fresh guava juice in one school in Cacaras [7] due to poor hygiene measures in the preparation of the fruit juice.

A salient feature of *T. cruzi* is extensive genetic heterogeneity [8–11]. The species is currently subdivided into six genetically discrete typing units (DTUs), TcI to TcVI [12, 13], and an additional clade associated with bats (TcBat) has been proposed [14]. TcI and TcII are ancient lineages that diverged from a common ancestor approximately 1–3 million years ago [10]. TcV and TcVI clearly have a relatively recent hybrid origin derived from TcII and TcIII [15]. According to some authors, TcIII and TcIV also originated from a more ancient hybridization of TcI and TcII [16, 17], although others claim that it is not the case [10, 18].

TcI is widespread from the southern USA to northern Argentina and Chile and infects many different mammal host including humans, domestic and sylvatic species, transmitted by triatomine bug vectors. TcI is the most frequently sampled DTU in wild transmission cycles, although it was also observed in domestic cycles [19, 20] and is the dominant DTU in terms of Chagas disease transmission in endemic regions north of the Amazon Basin [21]. In Brazil, TcI represents 58% of recovered sylvatic *T. cruzi* isolates [8]. Moreover, in Brazil, the distribution of DTUs does not appear to have an association with particular biomes or geographical areas [8]. Some species including bats and, in particular, marsupials (*Didelphis* spp.) are considered to be ancient reservoir hosts of *T. cruzi* [22]. Didelphids are nomadic sylvatic/synanthropic species and widely distributed throughout Brazil’s biomes, inhabiting both terrestrial and arboreal niches [8]. They are omnivorous and highly adaptable, capable of colonizing environments degraded by humans and are classically associated with the *T. cruzi* I genotype [23]. However, they are also able to harbor other *T. cruzi* DTUs. Hence, *Didelphis* spp. are exposed to *T. cruzi* infection across different transmission cycles and act as bioaccumulators of TcI intralineage genotypes [8].

The genetic diversity present in TcI has been assessed by a plethora of molecular methods including random amplification of polymorphic DNA (RAPD), multilocus enzyme electrophoresis (MLEE), internal transcribed spacer (ITS), and polymorphisms in minicircles and in the miniexon gene [23–28]. Several studies based on the microsatellite motif of spliced ladder intergenic regions (SL-IR) indicate the existence of five groups within TcI (Ia-Ie) and potential associations to anthropogenic or wild environments [16, 28–32]. However, a review of the SL-IR classification of five subgroups of TcI showed that there was no conclusive evidence of genetic structuring between domestic and wild isolates, but rather an association with geographical distribution [33, 34]. Ramirez et al. [35], using nuclear gene targets and MLST, demonstrated the existence of two TcI groups in Colombian isolates: one associated with the wild transmission cycle (TcI SILV) and the other with domestic transmission (TcI DOM). The latter has previously been classified as TcIa and TcI VEN_Dom_ [28, 36]. Ramirez & Hernandez [37] provided evidence for a possible subdivision of TcI (into DOM and SILV), although they also stated that the substancial diversity present in TcI did not allow conclusive identification of TcIDom as a robust near-clade.

MLST involves the sequencing of constitutive gene fragments. Initially applied to bacteria and yeast, and subsequently adapted and applied to diploid organisms, including *Trypanosoma cruzi* [38, 39] and *Leishmania* spp. [40]. The major advantage of MLST analysis is that sequence data are unequivocal with sufficient resolution for epidemiological diversity and population studies [38]. Furthermore, results are objective and easily accessible for some pathogens via online database repositories such as MLST.net [38]. Previously, and specific to *T cruzi*, Yeo et al. [38] applied MLST analysis using nine constitutive genes to evaluate the diversity across different DTU’s. Additionally, Lauthier et al. [39] assessed diversity on *T. cruzi*, using ten markers. Moreover, Messenger et al. [41] developed an MLST maxicircle typing scheme using ten gene targets applied to TcI isolates from disparate locations revealing extensive mitochondrial introgression and heteroplasmy. Furthermore, to analyze the variability of TcI in Colombia, Ramirez et al. [35], applied MLST analysis to 13 constitutive genes.

Brazil is a vast country, containing an enormous diversity of habitats and high biodiversity. Yet, little is known of the intra-DTU diversity of TcI in mammals, and more specifically in *Didelphis* spp., in Brazil. We investigate the potential for TcI subpopulation associations in the context of species of the marsupial genus *Didelphis* and their role in transmission cycles and as bioaccumulators of *T. cruzi*. We apply MLST analysis of four housekeeping genes, to assess the genetic heterogeneity of TcI isolates obtained from *Didelphis* spp*.* that were captured in four different Brazilian biomes spanning a vast geographical range.

## Methods

### Parasite isolates

**Fig. 1:**
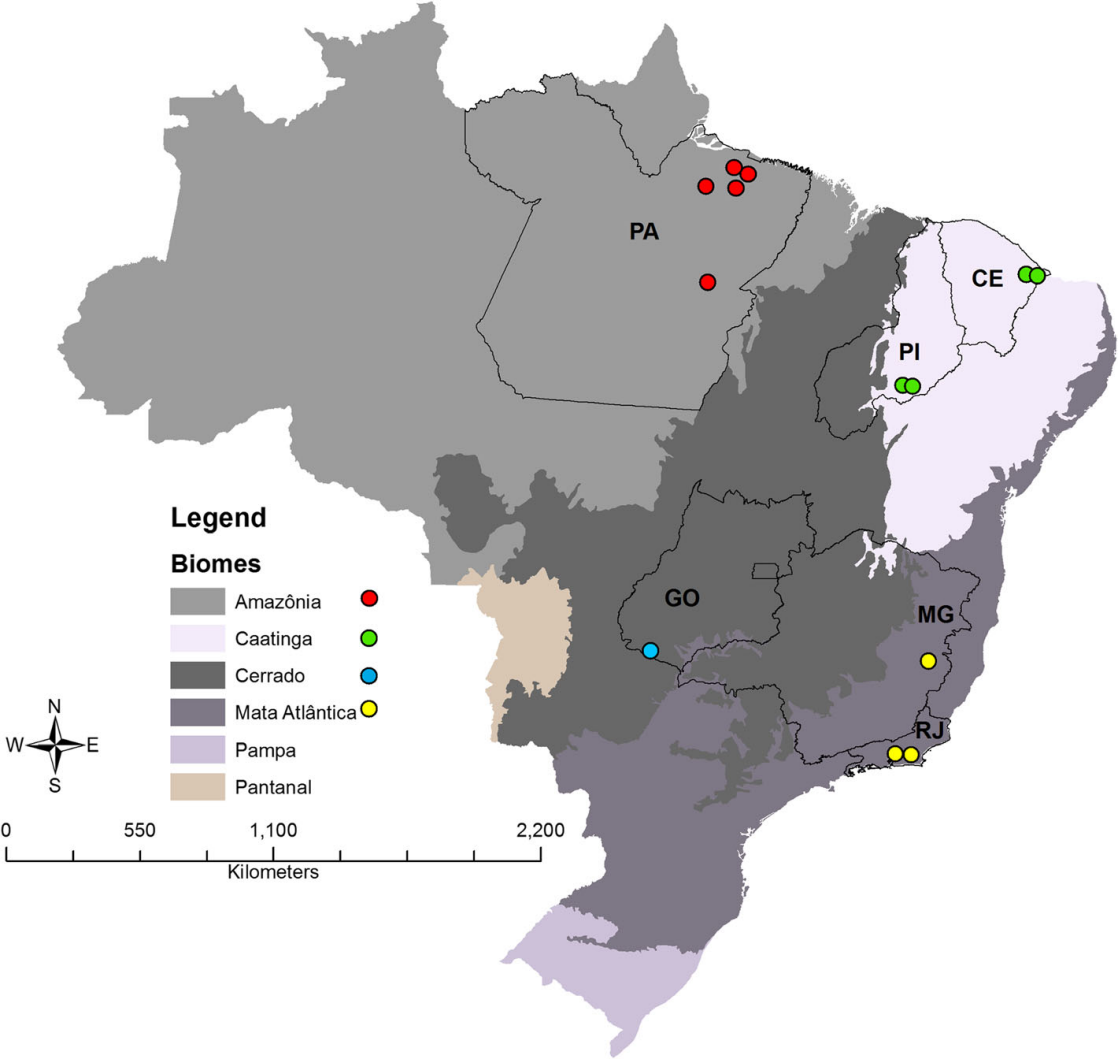
Map of the spatial distribution of the *T. cruzi* I isolates. Circles with colors represent the municipalities in each biome where samples were obtained: Amazon (red); Caatinga (green); Atlantic Forest (yellow); Cerrado (blue)

A total of twenty nine TcI isolates, available in the Coleção de Trypanosomas de mamiferos silvestres, domesticos e vetores **(**ColTryp) at the Laboratório de Biologia de Tripanosomatídeos do Instituto Oswaldo Cruz/FIOCRUZ, were sampled from *Didelphis marsupialis*, *D. albiventris* and *D. aurita*, captured in four Brazilian biomes: Atlantic Forest, Amazon, Caatinga and Cerrado (Fig. 1 and Table 1). TcI isolates that had previously been confirmed as TcI using Mini-Exon polymerase chain reaction (PCR) [42] by ColTryp, were genotyped by MLST with appropriate reference sequences.

**Table 1:** MLST: Characteristics of TcI isolates from *Didelphis* spp. from four Brazilian biomes

ID	Host	Biome	Municipality/ State	Year of collection	GenBank accession number			
					*DHFR-TS*	*RB19*	*LYT1*	*METIII*
D7	*D. aurita*	Atlantic Forest	Silva Jardim/ Rio de Janeiro	1996	MG228296	MG228324	MG228352	MG868974
G05	*D. marsupialis*	Atlantic Forest	Silva Jardim/ Rio de Janeiro	2003	MG228297	MG228325	MG228353	MG868975
G15	*D. marsupialis*	Atlantic Forest	Silva Jardim/ Rio de Janeiro	2003	MG228298	MG228326	MG228354	MG868976
G45	*D. marsupialis*	Atlantic Forest	Guapimirim/ Rio de Janeiro	2000	MG228300	MG228328	MG228356	MG868978
G41	*D. marsupialis*	Atlantic Forest	Silva Jardim/ Rio de Janeiro	2003	MG228299	MG228327	MG228355	MG868977
762	*D. aurita*	Atlantic Forest	Silva Jardim/ Rio de Janeiro	unknown	MG228290	MG228318	MG228346	MG868968
5574	*D. aurita*	Atlantic Forest	Capitão Andrade/ Minas Gerais	2003	MG228284	MG228312	MG228340	MG868962
12640	*D. marsupialis*	Amazonia	Abaetetuba/ Pará	2008	MG228280	MG228308	MG228336	MG868958
10272	*D. marsupialis*	Amazonia	Cachoeira do Arari/ Pará	2006	MG228276	MG228304	MG228332	MG868954
12625	*D. marsupialis*	Amazonia	Abaetetuba/ Pará	2008	MG228279	MG228307	MG228335	MG868957
10290	*D. marsupialis*	Amazonia	Cachoeira do Arari/ Pará	2006	MG228277	MG228305	MG228333	MG868955
6737	*D. marsupialis*	Amazonia	Itupiranga/ Pará	2004	MG228286	MG228314	MG228342	MG868964
6716	*D. marsupialis*	Amazonia	Itupiranga/ Pará	2004	MG228285	MG228313	MG228341	MG868963
12667	*D. marsupialis*	Amazonia	Curralinho/ Pará	2009	MG228281	MG228309	MG228337	MG868959
12668	*D. marsupialis*	Amazonia	Curralinho/ Para	2009	MG228282	MG228310	MG228338	MG868960
6824	*D. albiventris*	Caatinga	Jaguaruana/ Ceará	2004	MG228289	MG228317	MG228345	MG868967
M3	*D. albiventris*	Caatinga	Coronel José Dias/ Piauí	1998	MG228302	MG228330	MG228358	MG868980
8648	*D. albiventris*	Caatinga	Jaguaruana/ Ceará	2005	MG228293	MG228321	MG228349	MG868971
8622	*D. albiventris*	Caatinga	Jaguaruana/ Ceará	2005	MG228292	MG228320	MG228348	MG868970
11639	*D. albiventris*	Caatinga	Russas/ Ceará	2008	MG228278	MG228306	MG228334	MG868956
3510	*D. albiventris*	Caatinga	Jaguaruana/ Ceará	2001	MG228283	MG228311	MG228339	MG868961
6812	*D. albiventris*	Caatinga	Jaguaruana/ Ceará	2004	MG228287	MG228315	MG228343	MG868965
6813	*D. albiventris*	Caatinga	Jaguaruana/ Ceará	2004	MG228288	MG228316	MG228344	MG868966
M1	*D. albiventris*	Caatinga	Coronel José Dias/ Piauí	1998	MG228301	MG228329	MG228357	MG868979
10171	*D. albiventris*	Caatinga	São Raimundo Nonato/ Piauí	2006	MG228275	MG228303	MG228331	MG868953
9149	*D. albiventris*	Cerrado	Aporé/ Goiás	2006	MG228294	MG228322	MG228350	MG868972
9425	*D. albiventris*	Cerrado	Aporé/ Goiás	2006	MG228295	MG228323	MG228351	MG868973
8552	*D. albiventris*	Cerrado	Aporé/ Goiás	2005	MG228291	MG228319	MG228347	MG868969

Details of the origin and geographical distribution of the isolates are given in Table 1 and Fig. 1, respectively.

### MLST: Choice of loci

Four nuclear TcI gene fragments were selected for MLST analysis. The choice of targets was based on a previous work conducted by Yeo et al. [38] that showed the intralineage discriminatory capacity of 9 nuclear genes applied to *T. cruzi*. The four genes selected were: *DHFR-TS* (dihydrofolate reductase-thymidylate synthase); *RB19* (RNA-binding protein-19), *METIII* (metacyclin-III), and *LYT1* (lytic pathway protein).

### Molecular methods

PCRs were performed using different reaction conditions following Yeo et al. [38] with some modifications. For *DHFR-TS*, initial denaturation was at 94 °C for 3 min, followed by 30 cycles of 94 °C for 30 s, 58 °C for 1 min and 72 °C for 2 min. The cycle conditions for amplification of *RB19*, *METIII* and *LYT1* genes were: 94 °C for 3 min, followed by 30 cycles of 94 °C for 30 s, with an annealing temperature of 53 °C (*RB19*), 51 °C (*METIII*) or 57 °C (*LYT1*) for 30 s, and an extension of 72 °C for 45 s. All reactions had a final additional extension of 72 °C for 10 min. Each 25 μl total reaction volume contained: 50 ng genomic DNA, 0.2 μM of each primer, 2 mM of each dNTPs, 50 mM MgCl_2_ solution and 1 U *Taq* (BIO-21086, Bioline, London, UK). The products were visualized on agarose (2%) stained with ethidium bromide. PCR products were purified using Illustra GFX PCR DNA and Gel Band Purification Kits (GE Healthcare, Little, Chalfont, Buckinghamshire, UK). Bi-directional sequencing was performed using PCR primers and Big Dye Terminator Cycle Sequencing v.3.1 (Applied Biosystems, Foster city, CA, USA) in an ABI PRISM 3730 DNA Sequencer (Applied Biosystems) using standard protocols. Bio Edit v.7.0.4.1 [43] and DNASTAR Lasergene SeqMan v.7.0.0 programs were used to align and edit DNA sequences. Heterozygous positions were identified manually by the presence of two coincident peaks at the same locus (“split peaks”), verified in both forward and reverse directions and scored according to one-letter International Union of Pure and Applied Chemistry (IUPAC) nomenclature. All edited sequences were deposited in the GenBank database under the accession numbers MG228275–MG228358 and MG868953–MG868980 (Table 1).

### Data analyses for MLST

Gene sequences were analyzed to investigate intralineage diversity, evidence of recombination via phylogenetic incongruence at the level of individual diplotypes and haplotypes, and also to determine the best combination and minimum combination of loci that enable identification of the maximum number of diploid sequence types (DSTs). MLSTest software [44] was initially applied to calculate the typing efficiencies (TE) and discriminatory power (DP) for each target. TE is defined as the number of identified genotypes divided by the number of polymorphic sites within the target locus [45]. DP is defined as the probability that two strains are distinguishable when chosen at random from a population of unrelated strains [39].

*Trypanosoma cruzi* is a minimally diploid organism and as such heterozygosity renders MLST analysis more difficult than in haploid organisms. As mentioned above, heterozygosity was inferred from electropherograms by a double peak (two bases) at the same variable bi-allelic site [46]. One consequence of multiple bi-allelic sites is the presence of ambiguous allelic phases within loci and also ambiguous combinations of alleles across separate loci. However, it is possible for diploid sequence data (without phase resolution) to be concatenated across multiple loci [38, 45] and subsequently subjected to distance-based phylogenetic methods for the study of lineage assignment and of recombination. Here we apply an average state methodology described by Diosque et al. [45]. In more detail, the genetic distance between T and Y (heterozygosity composed of T and C) is considered as the mean distance between the T and the possible resolutions of Y (distance T-T =  0 and distance T-C = 1, average distance  = 0.5, see Diosque et al. [45] and MLSTest software [44] for further details.

Incongruence between phylogenetic trees derived from individual gene targets was analyzed via incongruence length difference (ILD) tests, in MLSTest software [44]. ILD tests evaluate differences between expected and observed incongruences between loci in the context of random unstructured homoplasy. All combinations from 2 to 4 fragments were analyzed using the optimization algorithm scheme in MLSTest which identifies the minimum combination of loci producing the maximum number of DSTs.

Phylogenetic analyses, for individual and concatenated genes sequences were performed using neighbor-joining (NJ) trees, implemented in MLSTest v.1.0 [44]. NJ trees were constructed using uncorrected p*-*distances, considering heterozygous sites as average states. One thousand bootstrap replications were used to evaluate branch support.

To infer haplotypes for each gene, diploid sequence data were analized using PHASE software v.2.1 [47]. This program is based on a modified Markov chain Monte Carlo (MCMC) algorithm which identified and assembles all unambiguous haplotypes. Bayesian phylogenetic analysis was subsequently performed (MrBayes), implemented through TOPALi v.2.5 [48], using the best-fit model selected, Hasegawa-Kishino-Yano plus gamma distributed rate variation among sites (HKY + G), based on the Bayesian information criterion. Five MCMC runs were carried out in parallel for one million generations with sampling every 100 generations and 25% burn in.

Reference sequences were obtained from LSHTM collaborators, from Yeo et al. [38] comprising TcI [C8 cl1, SAXP18 cl1, 9210601P cl1, PI (CJ007), PII (CJ005)] and also Tu18cl2 (TcII) and GenBank sequence (X10/1, CP015651.1). In addition to phylogenetic incongruence between single gene trees, analysis of allelic recombination to detect allelic gene mosaics at the level of individual genes was undertaken. Isolates with unambiguous phases were applied through the software package RDP3 (recombination detection program) [49], incorporating the following methods: RDP [50], Bootscanning [51], GENECONV [52], Maximum Chi Square method [53, 54], the Chimaera method [53], the Sister Scanning Method [55], the 3SEQ method [56].

## Results

### Genetic diversity and discriminatory power of MLST by diploid sequence typing

We observed a significant genetic diversity in the context of single nucleotide polymorphisms (SNPs), as well as differences and variations in discriminatory powers (DP) of the four constitutive gene fragments under study (Table 2).

**Table 2:** Measures of genetic diversity for 4 MLST housekeeping genes

Gene	No. of alleles	No. of polymorphisms	Typing efficiency (TE)	Discriminatory power (DP)
DHFR-TS	4	4	1	0.31
MET III	11	10	1.1	0.828
RB19	14	7	2	0.92
LYT1	14	14	1	0.89

For individual genes, the number of polymorphic sites ranged from 4 (*DHFR-TS*) to 14 (*LYT1*) and the associated number of alleles ranged from 4 (*DHFR-TS*) to 14 (*LYT1* and *RB19*). The most resolutive marker, i.e. distinguishing most genotypes, was *RB19* (TE = 2), which also possessed the highest discriminatory power (DP = 0.92). *LYT1* and *DHFR-TS* were the least resolutive genes (TE = 1), with *DHFR-TS* also possessing the lowest DP value of 0.31. The DP of 4 concatenated targets by MLST was 0.993, discriminating 33 out 35 isolates.

### Single locus phylogenies and MLST

Phylogenetic trees were generated for each marker (Additional file 1: Figure S1 and Additional file 2: Figure S2) to assess diversity and incongruence in topology. In more detail, *DHFR-TS* isolates formed a single cluster, indicative of limited variation, a finding in agreement with TE and DP figures for this target. All isolates grouped with reference sequences, with the exception of one isolate (8622) from Caatinga (Additional file 1: Figure S1). *RB19* revealed the presence of several clusters, although with relative low bootstrap support. Two clusters displayed bootstrap support > 50%: one containing 3 isolates from the Cerrado biome (9425, 8552, 9149), and the other cluster including 3 isolates from Amazonia (12667, 10272, 12625), one from the Atlantic Forest (G41) and 2 reference sequences (B187, X10*)*. *LYT1* produced a single cluster containing all 10 isolates from Caatinga (bootstrap value = 61%) (Additional file 2: Figure S2). Here again, 8552 and 9149 samples from Cerrado grouped together (bootstrap value = 64%). Within *LYT1*, reference sequences X10 (Bolivian *T. infestans*), C8 (Bolivian *T. infestans*) and SAXP18 (Peruvian *Didelphis marsupials*) clustered with moderate and high support (bootstrap values of 87 and 98%, respectively). The *METIII* tree (Additional file 1: Figure S1) supported a single cluster (bootstrap support > 58%) and grouped the sample 12668 from the Amazon region and 6812 from Caatinga with the reference sequences 9210160 (*Didelphis marsupials*, US) and X10, respectively.

Trees generated with *LYT1* and *RB19* (Additional file 2: Figure S2) and *METIII* (Additional file 1: Figure S1) genes, also exhibited topological inconsistencies. For example, within *LYT1* the subgroup containing all isolates from the Caatinga biome was not observed in other trees. The Atlantic Forest isolate G41 was similarly positioned in both *LYT1* and *METIII* topologies within Amazonian, Cerrado and Atlantic Forest isolates. In contrast, G41 was positioned in a well-defined cluster for *RB19* which contained isolates solely from the Amazon region (bootstrap value of 53%) (Additional file 2: Figure S2).

### Intra DTU I diversity

**Fig. 2:**
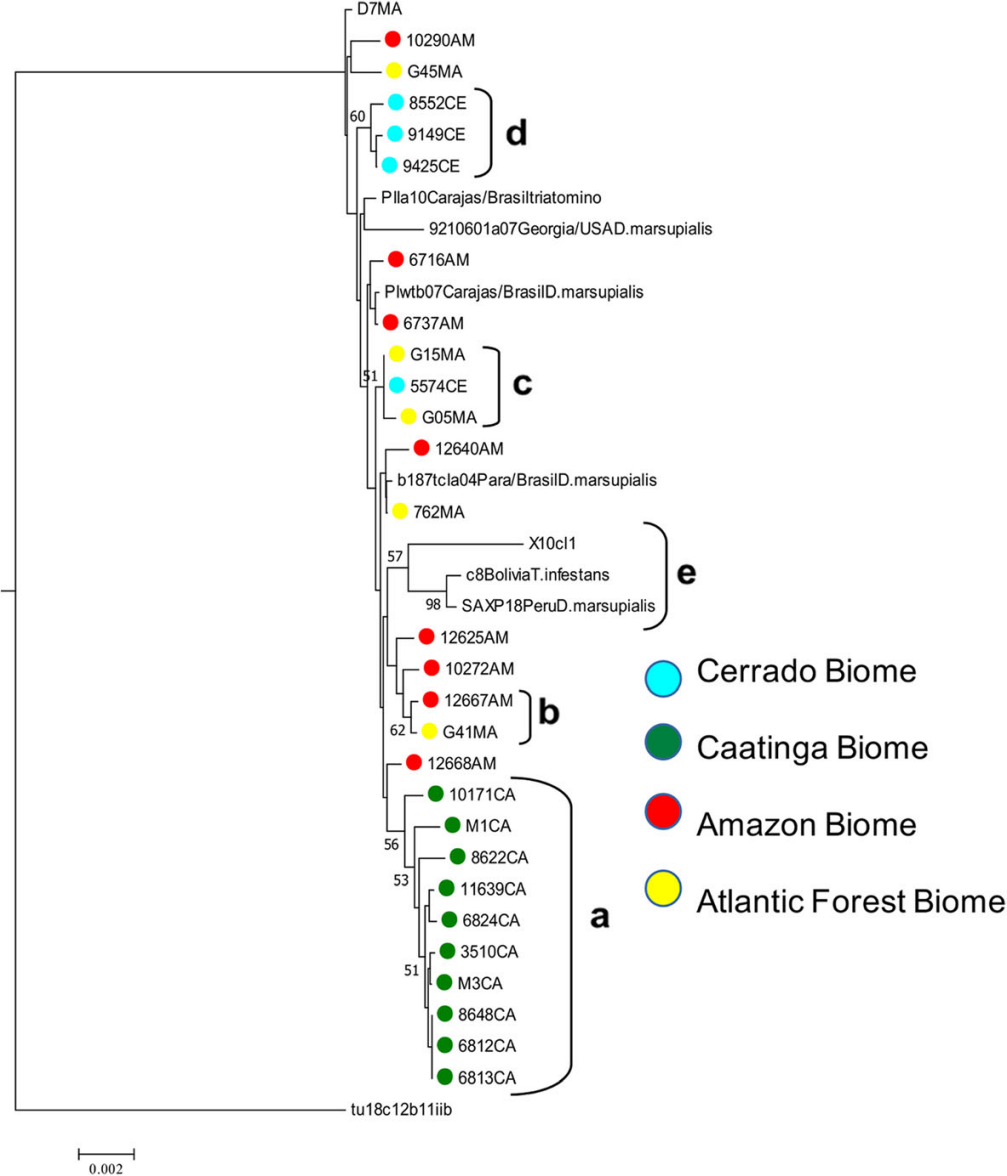
Neighbor-joining tree based on the concatenation of 4 MLST fragments. NJ trees were constructed using uncorrected p-distances and considering heterozygous sites as average states. Branch values represent bootstrap values (1000 replications). Circles with colors represent the municipalities in each biome where samples were obtained: Amazon (red); Caatinga (green); Atlantic Forest (yellow); Cerrado (blue)

The resolutive power for all four genes was assessed by concatenation to produce a single phylogeny. Generally, clusters consisted of a mixture of isolates from geographically disparate regions with the exception of the isolates originating from the Caatinga biome. In more detail, five clusters with moderate bootstrap support were observed (Fig. 2).

Cluster a (bootstrap value of 56%) exclusively included isolates from the Caatinga biome, as observed previously with *LYT1*. Cluster b (bootstrap value of 62%) included isolates from very distant regions, including the Atlantic Forest (G41 - Rio de Janeiro) and Amazonia (12667 - Curralinho), indicating the wide distribution of this TcI genotype. Cluster c (bootstrap value of 51%) grouped Atlantic Forest isolates (Rio de Janeiro) and a single isolate from Cerrado (Goias) further evidencing the wide distribution of the TcI genotypes (Fig. 1). Cluster d (bootstrap value of 60%) (Fig. 2) was comprised of remaining isolates from the Cerrado biome. Lastly, cluster e (bootstrap value of 57%) included the reference sequences X10, C8 and SAXP, as previously observed in the *LYT1* phylogeny.

**Fig. 3:**
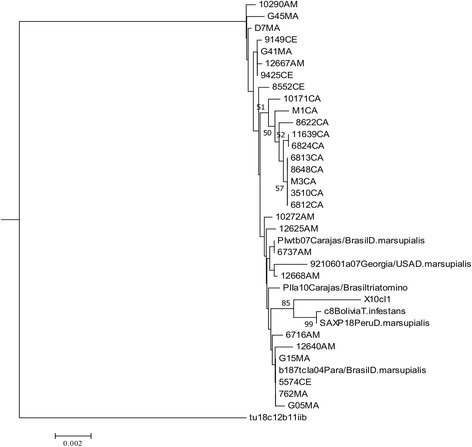
Neighbor-joining phylogenetic reconstruction using the three nuclear genetic markers (*DHFR-TS*, *LYT1* and *METIII*) concatenated*.* The tree was constructed using uncorrected p-distances and considering heterozygous sites as average states

Applying measures of incongruence across all combinations reveal the four fragment datasets are significantly incongruent (*P* < 0.05). However, on excluding *RB19* the *P*-value for ILD was not significant (BIONJ-ILD = 0.4) indicating that *DHFR-TS*, *LYT1* and *MET III* are broadly congruent (Fig. 3).

### Intralineage recombination

To further assess evidence for recombination, the allelic origins for each target were investigated. Haplotypes and associated phylogenies were generated for each of the three gene fragments (*LYT1*, *METIII* and *RB19*). Based on these haplotypes we observed evidence of genetic recombination among isolates in two genetic targets.

**Fig. 4:**
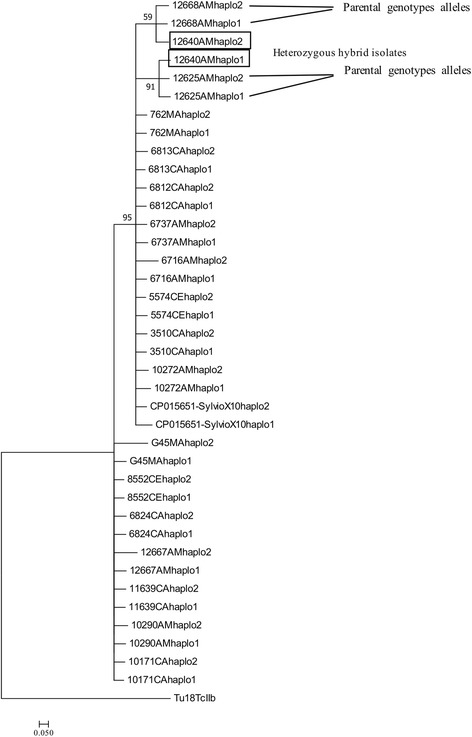
Haplotype neighbor-joining tree with *MET III* locus. The tree based on haplotypes with the program PHASE v.2.1. Black circles indicate heterozygous hybrid isolates (12640) and black line potential parental genotypes alleles (12625–12668)

The haplotypic trees for *METIII* revealed the putative homozygous donors genotypes, situated in different clusters and their corresponding heterozygous profiles (Fig. 4). Specifically, heterozygous isolate 12640 contained alleles that are consistent with putative donor haplotypes from homozygous isolates 12625 and 12668. Putative donor homozygous SNP profiles and the corresponding heterozygous profiles are presented in the Additional file 3: Table S1.

**Fig. 5:**
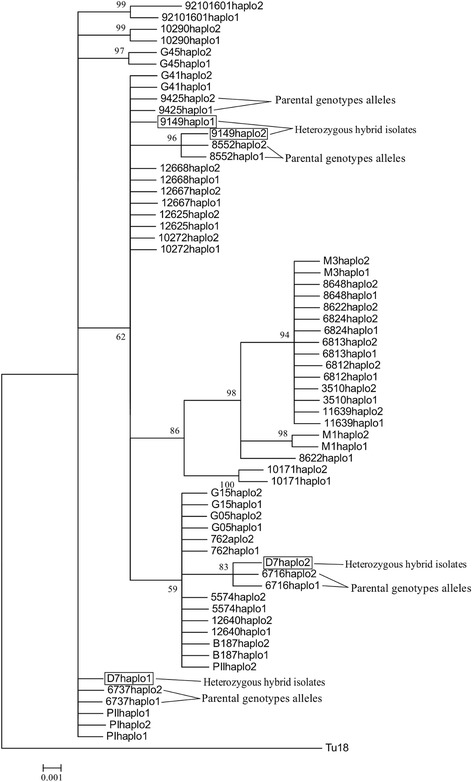
Haplotype neighbor-joining tree with *LYT1* gene**.** The tree based on haplotypes with the program PHASE v.2.1. Black circles indicate heterozygous hybrid isolates (9149 and D7) and black line potential parental genotypes alleles (8552-G05; 6737–6716)

Secondly *LYT1* demonstrated heterozygous haplotypes for isolate 9149 and D7, which clustered with its putative homozygous donors 8552 and 9425, and 6737 and 6716, respectively (Fig. 5). Putative donor-homozygous SNP profiles and the corresponding heterozygous profiles are presented in the Additional file 4: Table S2. Although this gives evidence of recombination at the level of allelic inheritance, for example through the segregation of alleles, we did not observe evidence of allelic recombination (mosaic alleles) through the RDP approach.

## Discussion

In this study, we evaluated the genetic diversity present in Brazilian TcI isolates, obtained from *Didelphis* spp*.* using an MLST approach. Our results confirmed the existence of significant genetic diversity within TcI in Brazilian didelphids [29, 32, 36]. Although we observed some evidence of geographical association in the Caatinga biome forming a single cluster (*LYT1* target) there was a general lack of population structure in association to any particular biome or habitat. Moreover, isolates originated from *Didelphis* spp. from geographically disparate areas and biomes possessed identical or similar genotypes.

MLST applied to 35 sequences (including reference sequences) indicated that the concatenation of the four gene fragments was highly discriminatory, identifying 33 out of 35 possible DSTs; however, a reduced panel of markers that included *DHFR-TS*, *METIII* and *LYT1* can discriminate 26 of 35 DSTs (Fig. 3). *LYT1* was the most polymorphic of the four fragments analyzed, in agreement with Ramirez et al. [36] and Yeo et al. [38]. However, a caveat to routine use of this target is the difficulty in optimizing reaction conditions to produce consistent quality sequence data, also observed by Yeo et al. [38].

*RB19*, with 7 polymorphic sites, possessed the highest typing efficiency (TE = 2), indicating that it is an excellent candidate for TcI diversity studies. Conversely, *DHFR-TS* was the least polymorphic. Yeo et al. [38] applied this marker for intralineage diversity studies finding it useful for discriminating DTUs TcVI and TcV. Despite the low number of SNPs we observed that the DP increased (0.25–0.31) with the inclusion of the *DHFR-TS*. However, in the context of a TcI specific cohort we suggest alternative, more discriminatory markers to *DHFR-TS*, be considered.

We also observed incongruences between phylogenetic trees, constructed with individual gene fragments, one explanation for which would be genetic recombination. Our data showed that isolates appeared in different clusters in the individual trees (Additional file 1: Figure S1 and Additional file 2: Figure S2). The existence of incongruences between constitutive gene trees has previously been observed in different lineages [19, 40, 45, 57] and it is considered a marker of populations that have undergone genetic recombination. The evidence for recombination is further supported by haplotypic phylogenies that infer allelic inheritance from homozygous donor genotypes. Importantly, for *MET III* and *LYT1*, heterozygous isolates were observed with their corresponding homozygous SNP donor genotypes (Additional file 3: Table S1 and Additional file 4: Table S2). We also examined haplotype sequences using various recombination detection algorithms (through RDP3), applied to individual alleles; we found no evidence of mosaics. As already observed by Yeo et al. [38], this result is not unexpected as the allelic recombination (mosaics) may be a rare event in comparison to inheritance by segregation of allelic haplotypes from and homozygous parental donors [38, 46]. TcV and TcVI, known recombinants of TcII and TcIII, were originally characterized by SNP distribution, in this way. Recombination in Tcl has been proven experimentally in a landmark publication [58], and in natural populations [20, 35, 41]. The frequency of recombination is unknown and may be common [41, 59] or rare [60, 61]. To obtain more refined results in the future, it would be important to include a much larger cohort of isolates with further cloning for downstream recombination analysis.

Concatenation of the four genes resulted in clustering all Caatinga isolates and excluded isolates from the other biomes, suggesting a geographical association at some level. It is worth noting that the area in question is rather extended, e.g. the municipalities of Jaguaruana and Sao Reimundo Nonato are separated by a distance of nearly 900 km (much more than the typical hosts displacement) and numerous barriers to hosts movement, including cities and roads [62]. The presence of similar genotypes over a wide area is in agreement with previous studies [63].

We generally observed similar genotypes spanning vast geographical distances, which is likely indicative of host/vector dispersion or clonal propagation over time against a background of intermittent recombination. As a further case in point, genetically similar genotypes were also present in both Atlantic Forest and Amazonia (cluster b, Fig. 2) forming a discrete subcluster, despite the corresponding localities being separated by a huge geographical distance. Amazonian isolates 12625 (Abaetetuba) and 10272 (Cachoeira do Arari) were clustered together despite their localities being separated by Bahia de Marajo, providing further evidence that similar genotypes span substantial distances across geographical barriers (Fig. 2). From our data, it is clear that different genotypes circulate sympatrically and infect multiple *Didelphis* spp. Of note, some of the localities, such as Abaetetuba and Curralinho in the state of Para, correspond to outbreak areas of Chagas disease [64]. Noticeably, Abaetetuba (Amazonian 12640, 12625) has had several outbreaks of Chagas disease reported [65]. Moreover, Llewellyn et al. [36] and also Ramirez et al. [35] identified specific genotypes involved in domestic and sylvatic transmission cycles in Venezuela (by microsatellites) and Colombia (by MLST), respectively. However, to investigate this possibility in Brazil a larger cohort of isolates originated from vectors, mammals, sylvatic and domestic isolates would be required.

We also observed local genotypic diversity as all isolates from the Cerrado biome (*n* = 4) were obtained from the same municipality (Apore), yet, only three of them grouped in the same cluster indicating localized diversity (Fig. 2). This phenomenon was observed by Lima et al. [63] who also detected extensive genetic diversity, in mainly sylvatic hosts, by the application of microsatellite analysis to isolates from the Cerrado biome (Tocantins), in which case these isolates clustered with isolates from the Atlantic Forest. Hence, the recurring theme from TcI in didelphids, presented here and also from also other TcI studies in Brazil, is one of extensive diversity but also with genetically similar isolates being present in disparate regions.

The presence of multiclonality in single hosts is known to occur [60, 66] and is a potential confounder for the genetic analysis from DNA from uncloned isolates. However, evidence from this particular cohort suggests that multiclonality can be ruled out as an explanation of the observed results. Specifically, as stated above, RDP analysis testing for recombination at the level of alleles (intra-allelic recombination) did not detect allelic mosaics in the clonal controls or the field isolates, which would likely be observed in the presence of mixed clones in individual isolates. Moreover, the evidence for recombination is derived from discrete allelic contributions from potential donor homozygous genotypes (and not allelic mosaics), further supporting the case for genetically discrete isolates over that of admixtures. Finally, these data show that similar genotypes span huge geographical distances. This is also suggestive of genetically discrete isolates and not admixtures of different clones. Together these results provide robust evidence for genetic diversity between discrete isolates within TcI.

The main epidemiological implication of our findings, is that there is no observed association between the distribution of subpopulations of TcI and the appearance of outbreaks of CD. Moreover, in Brazil, TcI has been classically associated with the sylvatic transmission cycle. This is, in part, due to its high prevalence in mammals and its high dispersion [42]. However, TcI has also been detected in human cases [67], in which it originated from sylvatic mammals including *Didelphis* spp. These marsupials are found in all forest strata in all biomes of Brazil. Additionally, they are known to dwell habitats in the proximity of human activity. These facts, together with their ability to harbour diverse TcI genotypes as seen in this study, clearly imply that understanding the role of the didelphids is of central importance to a more thorough elucidation of the TcI transmission cycles in Brazil.

## Conclusions

We conducted a MLST study using four nuclear genes applied to a panel of TcI isolates obtained from three didelphid hosts and spanning four ecologically disparate Brazilian biomes. Our results revealed considerable intra DTU genetic diversity using a sensitive panel of MLST markers and demonstrated a lack of clear associations of TcI genotypes to geographical location or transmission cycle. The one exception are isolates from Caatinga which clustered together. These data suggest that multiple TcI genotypes circulate sympatrically in mammalian host species, transmission cycles and probably insect vectors. We also infered the presence of intralineage genetic recombination by SNP distribution patterns and phylogenies at two loci (*METIII* and *LTY1)* in two isolates derived from *D. marsupialis* and *D. albiventris*, thus substantiating the important role of didelphids in TcI transmission in Brazil.

## Additional files

Additional file 1 **Figure S1** Phylogenetic incongruence between individual nuclear markers applied to 35 TcI Brazilian isolates. a NJ phylogenetic reconstruction using *DHFR-TS*. b NJ phylogenetic reconstruction using *METIII*. (PDF 122 kb)

Additional file 2 **Figure S2** Phylogenetic incongruence between individual nuclear markers applied to 35 TcI Brazilian isolates. a NJ phylogenetic reconstruction using *LYT1*. b NJ phylogenetic reconstruction using *RB19*. (PDF 122 kb)

Additional file 3 **Table S1** SNP data showing putative donor and recipient isolates for *METIII*. Sequences containing heterozygous SNPs (R) and putative homozygous donor (D) genotypes. (XLSX 12 kb)

Additional file 4 **Table S2** SNP data showing putative donor and recipient isolates for *LYT1*. Sequences containing heterozygous SNPs (R) and putative homozygous donor (D) genotypes. (XLSX 8 kb)

## Abbreviations

aCD: acute Chagas disease
ColTryp: Coleção de Trypanosomas de mamiferos silvestres, domesticos e vetores
*DHFR-TS*: Dihydrofolate reductase-thymidylate synthase
DP: Discrimination power
DST: Diploid sequence types
DTU: Discrete typing units
ILD: Incongruence length difference
ITS: Internal transcribed spacer
*LYT1*: Lytic pathway protein
MCMC: Markov chain -Monte Carlo
*METIII*: Metacyclin-III
MLEE: Multilocus enzyme electrophoresis
MLST: Multilocus sequence typing
NJ: Neighbor-joining
PCR: Polymerase chain reaction
RAPD: Random amplification of polymorphic DNA
*RB19*: RNA-binding protein-19
RDP: Recombination detection program
SL-IR: Spliced lader intergenic region
SNP: Single nucleotide polymorphism
TcI: *Trypanosoma cruzi* I
TE: Typing efficiency
