# Supplementary material for: Multilocus sequence typing: genetic diversity in Trypanosoma cruzi I (TcI) isolates from Brazilian didelphids
Source: Parasit Vectors. 2018 Feb 22;11:107. doi: 10.1186/s13071-018-2696-9 (PMC5824584; doi:10.1186/s13071-018-2696-9)

Additional file 2: Figure S2: Phylogenetic incongruence between individual nuclear markers applied to 29 TcI Brazilian isolates. **a**: NJ phylogenetic reconstruction using *LYT1***.** **b**: NJ phylogenetic reconstruction using *RB19*.

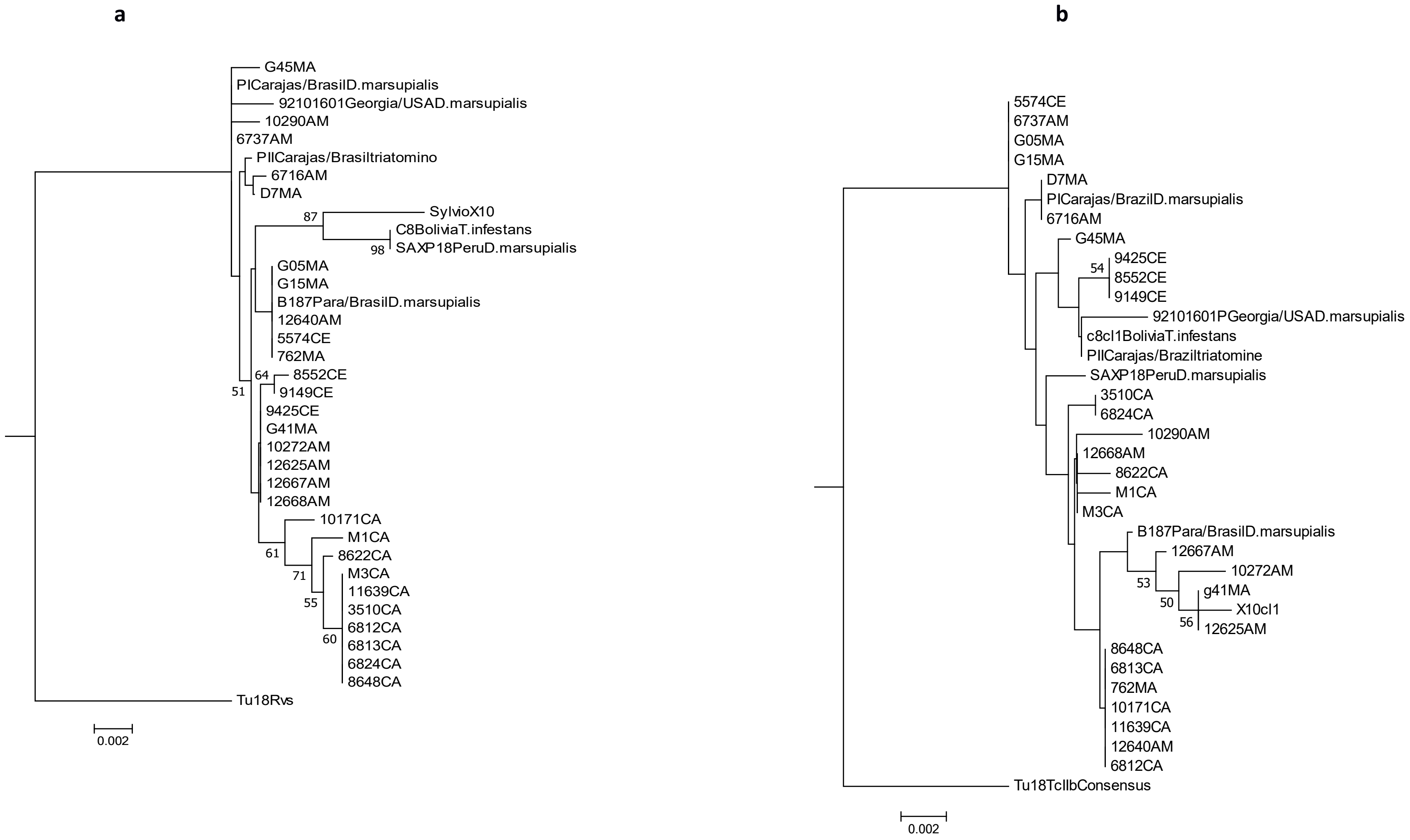

Supplement: Supplementary file 2 — Figure S2 Phylogenetic incongruence between individual nuclear markers applied to 35 TcI Brazilian isolates. a NJ phylogenetic reconstruction using LYT1. b NJ phylogenetic reconstruction using RB19. (PDF 122 kb) [file 13071_2018_2696_MOESM2_ESM.pdf]
